# The effectiveness of motivational interviewing on self-management in older adults with hypertension and frailty: study protocol for a randomized controlled trial

**DOI:** 10.3389/fpubh.2026.1850484

**Published:** 2026-06-17

**Authors:** Shihui Jia, Xinxin Wang, Xue Wang, Lishuang Zheng, Ranran Wang, Yueru Qi, Tianliang Ji, Guichen Li

**Affiliations:** 1 School of Nursing, Jilin University, Changchun, China; 2 The First Hospital of Jilin University, Changchun, China; 3 School of Nursing, The Hong Kong Polytechnic University, Kowloon, Hong Kong SAR, China

**Keywords:** frailty, hypertension, motivational interviewing, older adults, randomized controlled trial, self-management

## Abstract

**Background:**

Frailty and hypertension frequently coexist in older adults, posing substantial challenges to self-management and increasing the risk of adverse outcomes. Standard health education has shown limited effects in this population, and motivational interviewing may support sustained behavior change, yet evidence from randomized controlled trials remains scarce.

**Methods:**

This prospective, single-blind, parallel-group randomized controlled trial aims to evaluate the effectiveness of a Transtheoretical Model-based motivational interviewing intervention on self-management behaviors and related clinical outcomes in community-dwelling older adults with hypertension and frailty. A total of 72 participants will be recruited in Changchun, China, and randomized 1:1 to receive a 12-week Transtheoretical Model-based motivational interviewing intervention or usual health education. The primary outcome is hypertension self-management behavior, secondary outcomes include blood pressure, frailty status, quality of life, and safety outcomes assessed at five time points over 36 weeks. Intention-to-treat analysis will serve as the primary analytical strategy.

**Discussion:**

We hypothesize that the intervention will significantly improve self-management behaviors and produce favorable changes in blood pressure, frailty status, and quality of life. This study will provide practical guidance for self-management in older adults with hypertension and frailty, clarify the value of motivational interviewing in supporting sustained behavior change, and inform the development of more targeted intervention strategies for this population in community settings.

**Clinical trial registration:**

Identifier ChiCTR2500114706.

## 1 Introduction

As global population aging continues to accelerate (1), the burden of chronic diseases among older adults continues to rise, and healthcare needs have grown substantially. Consequently, maintaining the health and well-being of older adults has emerged as a pressing public health priority (2). Hypertension is one of the most common chronic diseases among older adults. It is defined as persistently elevated blood pressure, specifically systolic blood pressure (SBP) ≥ 140 mmHg and/or diastolic blood pressure (DBP) ≥ 90 mmHg on at least three separate occasions on different days, in the absence of antihypertensive medication (3). It is estimated that approximately 65% of adults aged 60–79 years and about 80% of those aged≥80 years have hypertension, and the total number of older adults living with hypertension is projected to increase from around 595 million in 2015 to approximately 1.41 billion by 2050 (4). Hypertension has now become the leading risk factor for cardiovascular disease and premature death worldwide (4). Frailty is an age-related geriatric syndrome characterized by reduced reserves across multiple physiological systems and diminished resistance to stressors, resulting in increased vulnerability to dependence and mortality (5). A global systematic review and meta-analysis reported a pooled prevalence of physical frailty of 12% in older adults (6). Frailty among older adults is closely associated with increased risks of adverse outcomes, including falls (7), disability (8), depression (9).

Given shared pathophysiological mechanisms, including chronic inflammation and oxidative stress, hypertension and frailty frequently coexist among older adults (10). A systematic review and meta-analysis reported that the prevalence of hypertension was 72% among frail older adults, while the prevalence of frailty was 14% among older adults with hypertension (11). Compared with their non-frail counterparts, older adults with hypertension and frailty are more likely to experience poorer quality of life, higher rates of rehospitalization, and increased mortality (12–18). The 2019 and 2023 Chinese Guidelines for the Management of Hypertension in Older Adults emphasize the importance of frailty management in the treatment of older patients with hypertension (19, 20). The management of older adults with hypertension and frailty is receiving increasing attention. Guidelines for hypertension recommend older adults with hypertension and frailty to be initially treated with monotherapy (20). The study by Bromfield et al. (21) indicated that older adults with hypertension and comorbid frailty have a significantly increased risk of falls. The study by Uchmanowicz et al. (22) demonstrated that the coexistence of frailty syndrome is associated with significantly poorer treatment adherence in older adults with hypertension, with an overall adherence rate of only 36.14% in their cohort. Older adults with hypertension and frailty face substantial challenges in self-management. Therefore, improving self-management in this population is of critical clinical importance.

Studies have indicated that health education serves as an important intervention strategy for the self-management of older adults with hypertension. The study by Bush et al. (23) indicated that health education interventions can improve blood pressure control among community-dwelling older adults with hypertension. Foroumandi et al. (24) demonstrated through a systematic review and meta-analysis that self-management health education programs targeting hypertensive patients can effectively enhance their self-efficacy. However, traditional health education typically follows a unidirectional delivery model, where healthcare providers transmit information and behavioral instructions to patients without sustained interaction or feedback (25). This approach results in low patient engagement, limited effectiveness, and difficulty in promoting meaningful health behavior change. Furthermore, providing health information alone often fails to promote sustained behavior change, especially among frail older adults whose health behaviors are closely influenced by motivational factors (26). These findings suggest that interventions predominantly reliant on external guidance are insufficient to meaningfully improve self-management. Instead, enhancing individuals’ intrinsic motivation for change is crucial to promote lasting behavioral modification. Motivational interviewing (MI) was first introduced by the American psychologist William R. Miller in 1983 and is defined as a client-centered, directive method for eliciting intrinsic motivation to change behavior by exploring and resolving ambivalence (27). MI has the advantage of enhancing intrinsic motivation, thereby facilitating sustained behavior change. A randomized controlled trial reported that motivational interviewing improved self-management capacity among older adults with chronic conditions (28). In another study, motivational interviewing was found to support blood pressure control and improve adherence among patients with hypertension (29).

The most effective educational approaches are theory-driven practices (30). The transtheoretical model (TTM) is recognized as a dynamic and comprehensive framework for understanding behavior change. It delineates the process into five distinct stages, and delivering stage-matched interventions has been extensively applied to facilitate the adoption of health behaviors (31, 32). The systematic review by Barzegar et al. demonstrated that interventions based on the TTM are effective in improving self-care behaviors in adults with hypertension, indicating its utility in promoting behavior change for better self-management (33). In the present study, the TTM is used to identify each participant’s current stage of change, which directly guides the selection of stage-matched counseling goals and strategies (34). The interventionist then applies motivational interviewing techniques, including open-ended questions, affirmation, reflective listening, and summarizing, to deliver these strategies in a collaborative and person-centered manner, eliciting change talk and resolving ambivalence (35). This integration ensures that intervention content is tailored to where each participant actually is in the change process, while the motivational interviewing approach supports their willingness to move forward, thereby promoting sustained behavior change (36). However, no randomized controlled trial has yet evaluated TTM-guided MI for self-management in older adults with comorbid hypertension and frailty. Therefore, guided by the TTM, we will conduct a randomized controlled trial to evaluate the effectiveness and feasibility of a motivational interviewing intervention aimed at improving self-management in older adults with hypertension and frailty.

We hypothesize that compared with usual health education, a 12-week TTM-based MI intervention will significantly improve hypertension self-management behaviors and produce favorable changes in blood pressure, frailty status, and health-related quality of life in community-dwelling older adults with hypertension and frailty. This study will generate evidence to inform strategies for supporting lasting health behavior change in this population.

## 2 Methods

### 2.1 Study design and aim

We will conduct a single blind, parallel randomized controlled trial (RCT). Such a design is supported by the Medical Research Council’s guidance for evaluating complex interventions (37). The protocol was developed in accordance with the CONsolidated Standards Of Reporting Trials (CONSORT) statement (38), and reported according to the Standard Protocol Items: Recommendations for Interventional Trials (SPIRIT) statement (39). This study aims to evaluate the effectiveness of a transtheoretical model based motivational interviewing intervention in improving self-management behaviors and clinical outcomes among older adults with hypertension and frailty.

### 2.2 Setting, recruitment, and eligibility

Participants will be recruited from two community health service centers (CHSCs) in Changchun, Jilin Province, China. Recruitment will take place over a planned period of 3 months, and the full trial is expected to be completed within 18 months from initiation. CHSCs serve as the primary setting for hypertension management in the Chinese community healthcare system, making them an appropriate venue for this intervention. To prevent contamination, the two CHSCs will be requested to stagger their routine health education activities. Participants will be recruited through live lectures and poster promotion. Eligible subjects will be screened via face-to-face surveys.

**Figure 1:**
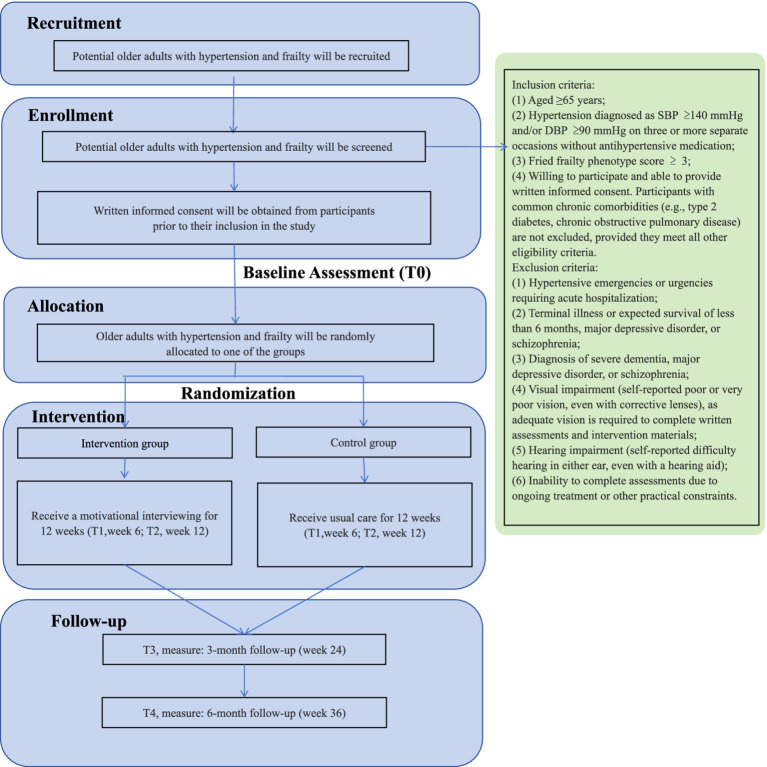
Study flow diagram of participant recruitment and intervention implementation.

Inclusion criteria: (1) aged ≥65 years; (2) hypertension diagnosed as SBP ≥ 140 mmHg and/or DBP ≥ 90 mmHg on three or more separate occasions without antihypertensive medication; (3) Fried frailty phenotype score ≥3; and (4) willing to participate and able to provide written informed consent. Participants with common chronic comorbidities (e.g., type 2 diabetes, chronic obstructive pulmonary disease) are not excluded, provided they meet all other eligibility criteria. Exclusion criteria: (1) hypertensive emergencies or urgencies requiring acute hospitalization; (2) terminal illness or expected survival of less than 6 months; (3) diagnosis of severe dementia, major depressive disorder, or schizophrenia; (4) visual impairment (self-reported poor or very poor vision, even with corrective lenses), as adequate vision is required to complete written assessments and intervention materials; (5) hearing impairment (self-reported difficulty hearing in either ear, even with a hearing aid); and (6) inability to complete assessments due to ongoing treatment or other practical constraints. The study flow diagram is presented in Figure 1.

### 2.3 Randomization, allocation, and blinding

Participants will be randomly assigned to either the intervention or control group in a 1:1 ratio using computer-generated permuted block randomization. An independent statistician will generate the allocation sequence. To ensure concealment, group assignments will be placed in sequentially numbered, opaque, sealed envelopes. Outcome assessors, data analysts, and study personnel involved in participant management will remain blinded to group assignment throughout the trial.

### 2.4 Intervention group

The intervention was developed based on evidence from the literature and refined through expert panel discussions. A pilot study was then conducted with older adults with coexisting hypertension and frailty to test comprehensibility and feasibility, and feedback was reviewed by the expert panel to further refine the intervention.

In the intervention group, participants will receive stage-matched motivational interviewing over 12 weeks, with sessions delivered every 2 weeks. Before each session, the participant’s current stage of change will be formally re-assessed using standardized questions, and counseling content, goals, and strategies will be tailored accordingly. MI techniques, including open-ended questions, affirmation, reflective listening, and summarizing, will then be applied to deliver stage-matched content in a collaborative and person-centered manner, eliciting change talk and resolving ambivalence. Each session will follow a structured format, approximately 5 min for stage re-assessment, 20–25 min for stage-matched MI counseling, and 5–10 min for goal-setting and session summarization. Each session will last 30–45 min. The intervention will cover six self-management domains: blood pressure control, medication management, diet and nutrition, physical activity, psychological and social support, and daily lifestyle, as summarized in Table 1. Full operational details are provided in Supplementary Table A2.

**Table 1:** Summary of the intervention components.

Stage	Content
Precontemplation stage	Blood pressure control: Assess symptom attributions and elicit perceived links with blood pressure fluctuations. Medication management: Assess necessity beliefs and concerns, and elicit misconceptions to address with empathic safety information. Diet and nutrition: Assess dietary patterns and elicit perceived links between diet and daily symptoms such as elevated blood pressure or physical discomfort. Physical activity: Assess exercise-related concerns and elicit views on the long-term impacts of physical inactivity on frailty progression and quality of life. Psychological and social support: Assess anxiety and depressive symptoms, and elicit awareness that emotional fluctuations may worsen blood pressure instability and frailty. Daily lifestyle: Assess smoking, alcohol use, and sleep problems, and elicit contextual reasons and triggers underlying these behaviors.
Contemplation stage	Blood pressure control: Use decisional balance to weigh the pros and cons of regular home blood pressure monitoring, and align targets with clinician guidance while avoiding overtreatment due to excessive blood pressure lowering. Medication management: Use decisional balance to link antihypertensive medication adherence with blood pressure stability and reduced risk of complications, and elicit patients’ own reasons for taking medication. Diet and nutrition: Use decisional balance to weigh the long-term benefits of a low-salt, low-fat diet for slowing frailty progression and improving quality of life. Physical activity: Use decisional balance to examine barriers and benefits of safe physical activity, and affirm any prior willingness or attempts to be active. Psychological and social support: Use decisional balance to examine how social support benefits mood, and elicit perceived advantages of actively seeking support. Daily lifestyle: Use decisional balance to compare changing versus maintaining smoking, alcohol, and sleep behaviors, focusing on fall prevention and preserving independence.
Preparation stage	Blood pressure control: Agree on an initial home monitoring frequency, standardize measurement conditions, and arrange regular outpatient reviews. Medication management: Support medication routines with practical reminders (pillbox or mobile phone alarms) and teach strategies to manage common side effects and adverse reactions. Diet and nutrition: Develop an individualized dietary plan (daily salt intake <6 g; five to six smaller meals per day). Physical activity: Develop a safe, progressive exercise program including warm-up, aerobic exercise (e.g., tai chi), resistance training (e.g., elastic bands), and balance training (e.g., tandem walking), progressing to at least 150 min per week, aligned with patient preferences. Psychological and social support: Develop an actionable social engagement plan, identify specific community activities or peer support resources, and schedule regular participation. Daily lifestyle: Develop specific behavioral plans (avoid screens 1 hour before bedtime; warm foot bathing; remove household tripping hazards; limit alcohol intake).
Action stage	Blood pressure control: Regularly review home blood pressure records and reinforce the link between health behaviors and outcomes. Medication management: When side effects or missed doses occur, collaborate on practical solutions and reinforce the importance of adherence for slowing frailty progression. Diet and nutrition: Affirm healthy food choices and provide practical strategies to address barriers in daily eating. Physical activity: Encourage recording exercise experiences; when weather or mood interferes, jointly adjust the plan and affirm efforts to maintain routine activity. Psychological and social support: Affirm social engagement attempts and explore integrating personal interests into daily life to manage anxiety. Daily lifestyle: Guide attention toward positive experiences and affirm efforts to sustain healthy daily routines.
Maintenance stage	Blood pressure control: Discuss sustaining home monitoring in special situations and encourage sharing effective strategies with family members. Medication management: Reinforce the importance of long-term medication adherence in older adults with hypertension and frailty. Diet and nutrition: Reframe occasional dietary lapses as learning opportunities, identify triggers, adjust strategies, and emphasize long-term benefits. Physical activity: To sustain long-term engagement, encourage diversifying activities within safety limits and summarize functional gains from sustained practice. Psychological and social support: Encourage role transition to enhance sense of purpose and motivation for continued participation. Daily lifestyle: Conduct monthly reviews to support long-term maintenance of healthy behaviors.

All sessions will be delivered by registered nurses who have completed a standardized MI training program. Ongoing supervision will be provided by a senior nurse with certified MI training throughout the trial period. To ensure intervention fidelity, all sessions will be audio-recorded with participant consent, and a random sample of 20% of recordings will be independently rated using the Motivational Interviewing Treatment Integrity (MITI) scale by a trained assessor blinded to outcome data (40). Regular supervision meetings will be held to review fidelity ratings and provide corrective feedback. Interventionists will maintain session logs documenting session duration, topics covered, and any protocol deviations.

### 2.5 Control group

Participants allocated to the control group will receive traditional health education, delivered through didactic lectures. Participants in the control group will receive a 12-week intervention delivered every 2 weeks, with each session lasting 30–45 min. The lecture content will be based on the finalized health education materials developed for this study (Supplementary Table A1).

### 2.6 Outcomes

#### 2.6.1 Outcome measures

Outcomes will be assessed by assessors blinded to group assignment at five pre-specified time points: baseline (T0, Week 0), mid-intervention (T1, Week 6), end of intervention (T2, Week 12), 3-month post-intervention follow-up (T3, Week 24), and 6-month post-intervention follow-up (T4, Week 36) (Table 2).

**Table 2:** Data collection schedule.

Measures	T0	T1	T2	T3	T4
Demographics	**△**				
HPSMBRS	**△**	**△**	**△**	**△**	**△**
BP	**△**	**△**	**△**	**△**	**△**
FP	**△**	**△**	**△**	**△**	**△**
SF-12	**△**	**△**	**△**	**△**	**△**
Safety monitoring		**△**	**△**	**△**	**△**

The measurement tools included (abbreviations): HPSMBRS, Hypertension Patients Self-Management Behavior Rating Scale; BP, Blood Pressure; FP, Frailty Phenotype; SF-12, 12-item Short-Form Health Survey; The symbol Δ indicates administration of the measure at the corresponding time point.

##### 2.6.1.1 Hypertension patients self-management behavior scale

Self-management behavior will be assessed using the Self-management Behavior Scale developed by Zhao et al. (41). The scale has demonstrated excellent internal consistency (Cronbach’s *α* = 0.914) and good validity (validity index = 0.910). It comprises 33 items across six domains: disease monitoring, diet management, work–rest management, exercise management, emotional management, and medication management. Items are rated on a 5-point Likert scale (1 = never, 2 = rarely, 3 = sometimes, 4 = often, 5 = always), yielding a total score ranging from 33 to 165, with higher scores indicating better self-management behavior.

##### 2.6.1.2 Blood pressure

Blood pressure will be measured in accordance with the blood pressure measurement protocol specified in the 2019 Chinese guidelines for the management of hypertension in older adults (20).

##### 2.6.1.3 Frailty phenotype

The frailty phenotype (FP) will be evaluated according to the widely used Fried criteria (5). The assessment comprises five components: unintentional weight loss, reduced handgrip strength, slow walking speed, self-reported exhaustion, and low physical activity level. A score of 1 is assigned for each criterion present, yielding a total score from 0 to 5. Based on this score, participants will be categorized as robust (0), pre-frail (1–2), or frail (≥3).

##### 2.6.1.4 12-item short-form health survey

Quality of life will be assessed using the 12-item short-form health survey, a shortened version of the 36-item short-form health survey, which has been widely used to evaluate health-related quality of life in both general and patient populations (42), The SF-12 yields two summary scores: the physical component summary and the mental component summary. Higher scores indicate better quality of life.

##### 2.6.1.5 Safety monitoring

The safety of all participants will be monitored throughout the trial. Any adverse health event occurring during or between intervention sessions will be recorded at each scheduled assessment time point and through spontaneous reporting by participants or their family members. Events of particular concern include falls, dizziness, hypotension, syncope, and acute cardiovascular or cerebrovascular events. Should any participant experience a life-threatening event or require emergency hospitalization, the research team will notify the Ethics Committee within 24 h and take immediate protective action, including temporary or permanent withdrawal from the trial if necessary. Safety data will be reviewed at regular intervals, and the trial may be suspended if an unexpected pattern of harm is identified.

#### 2.6.2 Adherence

To enhance participant adherence, we will clearly communicate the value of their contribution to the study and provide individualized reminders prior to each intervention session. In accordance with the spirit of motivational interviewing, the interventionist will actively listen to feedback and reserve sufficient time after each session for discussion and clarification of questions. Participation is entirely voluntary; participants may withdraw unconditionally at any time without affecting their entitled rights or benefits.

#### 2.6.3 Sample size and power calculations

An *a priori* power analysis was conducted using G*Power 3.1.9.7. Based on a similar intervention study by Tan (43), post-intervention self-management scores were 73.66 (SD = 5.55) in the intervention group and 68.26 (SD = 5.93) in the control group, yielding a pooled standard deviation of 5.74 and a Cohen’s d of 0.94. Using a two-sided independent samples comparison with *α* = 0.05 and power = 80% (1 − *β* = 0.80), the minimum required sample size was 30 participants per group. Considering the elevated risks of hospitalization, health deterioration, and mobility limitations in frail older adults, a conservative attrition rate of 15% was applied. The final sample size was therefore 36 per group (72 in total).

#### 2.6.4 Quality control

Our research team is an experienced multidisciplinary group comprising two gerontology experts, one psychologist, one nutrition specialist, one clinician, one clinical nurse, and three graduate students in nursing. The research team will deliver unified and standardized training to all personnel responsible for implementing the intervention to ensure consistent delivery and fidelity. If complex clinical issues arise, the researchers will collaborate with relevant specialist experts to jointly develop appropriate solutions.

#### 2.6.5 Statistical analysis

For quantitative analysis, Epidata 3.1 (Epidata Association) will be used for double data entry and SPSS 26.0 will be used for data analysis and processing. Descriptive statistics will be used to describe participants’ characteristics and mental health outcomes. For continuous variables, the Shapiro–Wilk test (S-W test) will be used to assess normality. Depending on the results of the S-W test, the mean and standard deviation or the median and the interquartile range will be used for descriptive statistics. For categorical variables, frequencies and percentages will be reported. Independent samples *t*-tests, Mann–Whitney U tests and χ^2^ tests will be used to compare outcome results and baseline data. Paired sample *t*-tests or non-parametric rank sum tests will be used to compare the outcomes within the group. To analyze between-group differences, a two-independent sample *t*-test or a Mann–Whitney U rank sum test will be used. The significance level of all the above statistical tests will be set at *p* ≤ 0.05, indicating a significant statistical difference. The primary analysis will follow the intention-to-treat principle, including all randomized participants regardless of protocol adherence or withdrawal. A per-protocol sensitivity analysis will also be conducted, restricted to participants who complete at least four of the six planned sessions (≥67%). Missing outcome data will be handled using multiple imputation under the missing-at-random assumption, with 20 imputed datasets pooled according to Rubin’s rules. Participants with complete data across all time points will additionally be analyzed as a further sensitivity check. Changes in primary and secondary outcomes across the five time points (T0–T4) will be analyzed using generalized estimating equations, accounting for the correlated structure of repeated measurements and adjusting for baseline differences. The primary test of differential change between groups will be the time-by-group interaction effect. All analyses will be conducted using SPSS 26.0.

#### 2.6.6 Ethical approval and trial registration

The study has been reviewed and approved by the Clinical Research Ethics Committee of the School of Nursing, Jilin University (Approval No. 2025112708), and has been registered in the Chinese Clinical Trial Registry (Registration No. ChiCTR2500114706) on 16 December 2025. The confidentiality and anonymity of participant data will be ensured throughout the entire study process, including during implementation of the trial protocol and in any subsequent reports or publications arising from this study. Prior to study initiation, a participant information sheet will be provided, and written informed consent will be obtained to ensure compliance with ethical standards. In addition, if any participant is identified as presenting with special symptoms during the study, our trained research team will conduct a prompt and comprehensive assessment and, when necessary, provide appropriate professional intervention.

## 3 Discussion

This trial is designed to evaluate whether a 12-week Transtheoretical Model-based motivational interviewing intervention can improve self-management behaviors in community-dwelling older adults with hypertension and frailty. We anticipate that the intervention group will demonstrate significantly greater improvements in hypertension self-management behavior scores compared with the control group at the end of the intervention and at post-intervention follow-up assessments, accompanied by favorable changes in blood pressure control, frailty indicators, and health-related quality of life.

Older adults with hypertension and frailty represent a population requiring complex, sustained care. Evidence on self-management in this population remains scarce, and studies have highlighted the need for clinical trials in this population. Benetos et al. (44) explicitly highlight an urgent need for controlled clinical trials focusing on the most frail older hypertensive patients, a population that has been systematically excluded from previous clinical trials, to establish stronger evidence regarding the benefits of various therapeutic strategies.

Unlike traditional health education, motivational interviewing employs a patient-centered, collaborative approach that elicits patients’ intrinsic motivation for change by exploring and resolving ambivalence, thereby supporting sustained health-related behaviors. Evidence also suggests that motivational interviewing is a valuable tool for primary healthcare nurses in health promotion practice (45). The transtheoretical model emphasizes matching stage-specific tasks and strategies and helps explain individual differences in behavior change (46). In this study, motivational interviewing is integrated with the transtheoretical model, with a key advantage of bridging the gap between knowledge and sustained behavioral action. If improvements in self-management behaviors are observed alongside better blood pressure control or favorable changes in frailty indicators, the findings will further support the proposed theoretical pathway in which enhanced motivation and stage progression lead to behavioral improvement and, in turn, benefits in health outcomes. If no significant effects are found, the results may indicate the need to refine stage assessment methods, optimize counseling language, adjust follow-up intensity, or improve goal-setting strategies. Throughout, this protocol seeks to balance feasibility with effectiveness.

The main anticipated implementation barriers are participant retention and outcome measurement. Frail older adults may have health fluctuations, acute hospitalization, or competing demands. These can interfere with session attendance. To reduce this risk, we will use flexible scheduling and proactive monthly contact. The primary outcome relies on self-reported measures. To minimize response bias, we will use standardized administration by trained and blinded assessors.

## 4 Conclusion

We hypothesize that the intervention will significantly improve self-management behaviors and produce favorable changes in blood pressure, frailty status, and quality of life. This study will provide practical guidance for self-management in older adults with hypertension and frailty, clarify the value of motivational interviewing in supporting sustained behavior change, and inform the development of more targeted intervention strategies for this population in community settings.
