# Supplementary material for: The effectiveness of motivational interviewing on self-management in older adults with hypertension and frailty: study protocol for a randomized controlled trial
Source: Front Public Health. 2026 Jun 17;14:1850484. doi: 10.3389/fpubh.2026.1850484 (PMC13322311; doi:10.3389/fpubh.2026.1850484)
Supplement: Supplementary file 1 [file Table_1.DOCX]

Supplementary Material

**Supplementary Table A1: Health education content**

The following content was delivered to the control group (usual health education) and also formed the informational basis for the intervention group’s self-management d

| Domain | Health Education Content |
| --- | --- |
| Blood pressure control | 1. For older adults with hypertension and frailty, antihypertensive therapy should be considered when BP ≥160/90 mmHg. The initial target is SBP <150 mmHg; if well tolerated, further reduction to <140/90 mmHg is acceptable. Avoid excessive BP lowering to reduce risk of cerebral hypoperfusion and falls. BP management should follow medical advice.   2. Blood pressure should be monitored regularly under standardized conditions, consistent timing, posture, measurement site, and the same validated sphygmomanometer whenever possible.  3. Long-term antihypertensive pharmacotherapy should be continued, pre-dose BP monitoring is recommended to evaluate treatment effectiveness. Even when BP is well controlled, follow-up visits every 1–3 months are advised. |
| Medication management | 1. At treatment initiation, monotherapy with a low-dose, long-acting antihypertensive agent is preferred. Participants should strictly follow medical advice and should not adjust doses, switch medications, or discontinue treatment independently. Misconceptions about medication use should be identified and corrected.   2. Participants should be educated on the correct medication regimen, potential adverse effects, and key precautions (e.g., changing posture slowly to avoid orthostatic hypotension and postprandial hypotension).  3. Participants should regularly review home medication supplies and check expiration dates. Reminder aids (e.g., pill organizers or alarms) may help reduce missed doses. |
| Diet and nutrition | 1.Dietary salt intake should be limited to <6 g/day (approximately one beer-bottle cap). Salt substitutes may be considered but should be avoided in participants with renal insufficiency.  2. A balanced diet should be encouraged, increased fresh fruits, vegetables, and whole grains, limited high-fat, high-cholesterol, and high-sugar foods, adequate high-quality protein (e.g., fish, eggs, dairy).  3. Dietary intake should follow principles of nutritional balance, variety, and easy digestibility. For participants with reduced appetite, 5–6 small, frequent meals per day are recommended to reduce gastrointestinal burden.  4. Food texture should be soft, finely chopped, and easy to swallow (e.g., minced meat, steamed eggs, or well-cooked noodles). Meal choices aligned with personal preferences support long-term adherence. |
| Physical activity | 1. A safe, progressive, multicomponent exercise program is recommended. Aerobic activity (e.g., slow walking, Tai Chi, Baduanjin) improves cardiorespiratory fitness; resistance training (e.g., resistance bands, 1–2 kg dumbbells) enhances muscle strength; balance training (e.g., heel-to-toe walking) reduces fall risk.   2. Exercise intensity should be moderate, light sweating, increased heart rate, but still able to talk comfortably. Begin with 5–10 min/session, gradually increasing to 20–30 min, target 150 min/week total, accumulated across multiple sessions. If 150 min/week is not feasible, any increase in activity above baseline is beneficial.  3. Warning signs requiring immediate cessation, chest pain, severe shortness of breath, dizziness, or blurred vision. If symptoms do not resolve with rest, prompt medical attention should be sought. |
| Psychological and social support | 1.Participants should be informed that negative emotions (e.g., anxiety, depressive mood) may contribute to BP fluctuations and accelerate frailty progression. Engaging in enjoyable activities (e.g., journaling, music, gardening, social conversation) is recommended to manage intense emotions.  2.Participants should be encouraged to share their health plan with family members to obtain practical support (medication reminders, meal preparation, accompaniment during exercise). Regular contact with friends, neighbors, or community activities reduces social isolation. |
| Daily lifestyle | 1.Complete smoking cessation is strongly recommended, secondhand smoke exposure should be avoided. If alcohol cannot be completely avoided, men <25 g/day (spirits ≤50 mL, wine ≤100 mL, or beer ≤300 mL), women <15 g/day.  2.Indoor temperature should be maintained ≥20°C with regular ventilation. During cold spells or strong winds, outdoor activities should be minimized; appropriate warm clothing (hat, scarf) should be worn when going outdoors.  3. Healthy sleep habits, dark, quiet bedroom, appropriate pillow height and mattress support; avoid screens 1 hour before bedtime, relaxing pre-sleep activities (warm foot soaking, calm music, reading).  4.Home safety, keep floors dry and obstacle-free, use non-slip bathroom mats, ensure adequate indoor lighting, and turn on lights when rising at night. |

|  |
| --- |

**Supplementary Table A2: Detail intervention protocol**

**A2.1 Intervention overview**

The intervention is a 12-week, theory-based motivational interviewing (MI) program grounded in the Transtheoretical Model (TTM). It consists of six individual face-to-face counseling sessions delivered by trained registered nurses at community health service centers in Changchun, China.

**Theoretical integration:** The TTM provides the structural scaffold for intervention delivery. Each participant’s stage of change (Precontemplation, Contemplation, Preparation, Action, or Maintenance) is formally re-assessed at the beginning of every session using standardized questions (see the TTM Stage Assessment Guide below). Stage-matched counseling content, goals, and strategies are then selected accordingly. MI provides the relational and communication methodology through which stage-appropriate content is delivered, using the OARS techniques (Open-ended questions, Affirmations, Reflective listening, and Summaries) to elicit change talk and resolve ambivalence. The TTM determines what content and goals are appropriate for the participant’s current stage. MI determines how the counselor communicates to support the participant in moving forward.

**A2.2 Standard session structure**

Each session follows the four-phase structure below. Total session duration is 30–45 minutes. Sessions are conducted at Weeks 1, 3, 5, 7, 9, and 11 of the 12-week intervention period.

| Session Phase | | Content | Key Activities / Techniques | Duration |
| --- | --- | --- | --- | --- |
| 1. Opening rapport | Greet participant; ask about general well-being; review progress on goals set at the previous session | | Open-ended questions; affirmation of any efforts or progress made; reflective listening | ~5 min |
| 1. TTM stage   re-assessment | Re-evaluate the participant’s current stage of change for the focal self-management domain(s) using standardized questions (see Stage Assessment Guide below) | | 2–3 standardized TTM staging questions; record stage on session log; adjust counseling plan accordingly | ~5 min |
| 3. Stage-matched MI counseling | Deliver stage-appropriate content across the six self-management domains using MI communication techniques (OARS); follow the domain‑specific protocol in Tables A2.6a–A2.6e | | Open-ended questions; Affirmations; Reflective listening (simple and complex); Summaries; Elicit change talk; Decisional balance (Contemplation); Action planning (Preparation/Action) | 20–25 min |
| 4. Goal-setting summary | Collaboratively set 1–2 specific, achievable behavior goals for the coming week; summarize key discussion points and affirm the participant’s strengths and intentions | | Collect-evoking summary; express confidence in participant’s ability | 5–10 min |
| Total session duration | 30–45 minutes per session × 6 sessions (Weeks 1, 3, 5, 7, 9, 11) | | | |

**A2.3 TTM stage assessment guide**

At the start of each session, the counselor uses the following standardized questions to identify the participant’s current stage of change for the primary self-management domain being addressed. Stages may shift between sessions; all subsequent counseling content is matched to the current stage identified.

| Stage | Operational Definition | Standardized Staging Question (asked at start of each session) | Counselor Response |
| --- | --- | --- | --- |
| Precontemplation | Not considering change in the next 6 months; unaware of or resistant to the need to change | “Have you ever thought about changing your (specific behavior, e.g., blood pressure monitoring habits)? Do you think it is important for you right now? AND “Are you planning to make any change in the next 6 months?” | Participant answers "no" to both intention and importance OR acknowledges importance but states no intention to change in next 6 months → Precontemplation |
| Contemplation | Aware of need to change; considering change within the next 6 months but not yet committed | “Are you thinking about making any changes to your (behavior) in the next few months? What makes you hesitate?” | Participant expresses ambivalence or weighs pros/cons; intends to change within 6 months but not within 30 days → Contemplation |
| Preparation | Intending to take action within the next 30 days; may have taken some small steps | “Are you planning to start (behavior) soon say, within the next month? Have you already tried anything? (e.g., bought a home BP monitor, tried reducing salt once)” | Participant expresses intention within 30 days and reports some preparatory steps (even if not fully consistent) → Preparation |
| Action | Has made the desired behavior change within the past 6 months | “Have you been doing (behavior) regularly? For how long have you been doing this?” | Behavior initiated but <6 months → Action |
| Maintenance | Has sustained the behavior change for more than 6 months | “You mentioned you have been doing (behavior) consistently. Is that something you have kept up for more than 6 months?” | Sustained >6 months → Maintenance |

**A2.4 MI feedback and response strategies**

The following table specifies how counselors should respond to common communication situations encountered during sessions. These strategies operationalize the MI spirit (partnership, acceptance, compassion, and evocation) and are applied across all TTM stages and self-management domains.

| Communication Situation | MI Technique | Example Response |
| --- | --- | --- |
| Participant expresses change talk (e.g., “I think I could try that”) | Simple reflection and Affirmation: immediately reinforce change talk to amplify motivation | “It sounds like you are already thinking seriously about this. That really shows how much you care about your health.” |
| Participant expresses sustain talk (e.g., “I feel fine as I am”) | Double-sided reflection: acknowledge both sides without confrontation or persuasion | “On one hand, things feel okay to you right now. On the other hand, you mentioned that you sometimes worry about your blood pressure going up. Both of those things make sense.” |
| Participant reports successful behavior (e.g., measured BP every day this week) | Specific affirmation: praise the specific effort, not general praise | “You measured your blood pressure every single day this week, even on the day you were feeling tired. That kind of consistency is exactly what makes a real difference.” |
| Participant reports a lapse or setback | Normalize and Reframe: frame lapse as a learning opportunity, not a failure | “It is very normal for this to happen most people find it takes several tries. What do you think got in the way? What could we do differently next time?” |
| Participant is silent or avoidant | Open-ended re-invitation: gently re-engage without filling the silence with advice | “Take your time there is no right or wrong answer. What comes to mind when you think about this?” |
| Participant resists or pushes back (e.g., “The nurse is wrong about this”) | Roll with resistance: avoid argumentation; redirect with curiosity | “It sounds like you have thought a lot about this and have some doubts. I am curious — what has your own experience been?” |

|  |
| --- |

**A2.5 Intervention fidelity**

**Interventionist qualifications:** All sessions will be delivered by registered nurses who have completed a standardized MI training program.

**Fidelity monitoring:** All sessions will be audio-recorded (with participant consent). A randomly selected 20% of recordings will be independently rated using the Motivational Interviewing Treatment Integrity (MITI) scale by a trained assessor blinded to outcome data. Regular supervision meetings will be held to review ratings and provide corrective feedback. Counselors will maintain session logs documenting session duration, topics covered, and any protocol deviations.

**A2.6 Stage by stage domain specific protocol**

The following five tables provide domain-specific counseling goals, representative open-ended questions, and key MI techniques for each TTM stage. Counselors should select and adapt questions based on the participant’s specific situation, preferences, and current concerns.

**Table A2.6a Precontemplation Stage**

Core goal: Raise awareness, build rapport, and plant seeds for reflection , without pressure to change. Avoid unsolicited advice.

| Domain | Core MI Goal | Representative Questions |
| --- | --- | --- |
| Blood pressure control | Raise awareness; explore personal meaning of BP symptoms | “Do you ever notice any symptoms that you think might be related to your blood pressure, such as headaches or dizziness?”  “What does having high blood pressure mean to you personally?”  “What would it mean for your daily life if your blood pressure were not well controlled?” |
| Medication management | Explore medication beliefs; address misconceptions without persuading | “How do you feel about taking blood pressure medication every day?”  “Some people have concerns about long-term medication. What are your thoughts on that?”  “What would help you feel more comfortable about the idea of taking medication regularly?” |
| Diet and nutrition | Enhance awareness of diet–health link; reduce defensiveness | “What does a typical day of eating look like for you?”  “Have you ever noticed any connection between what you eat and how you feel physically?”  “What do you enjoy most about your current eating habits? Are there any parts you have ever thought about adjusting?” |
| Physical activity | Explore concerns about exercise; build confidence in safety | “What does exercise mean to you right now? Does it feel like something that fits into your life?  “Some people your age worry about falls when they exercise. Do you have any concerns like that?”  “If you did do some physical activity, even just a little, what might that look like for you?” |
| Psychological and social support | Assess emotional and social context; introduce idea of connection | “How are you feeling in general these days, emotionally?”  “Do you have people around you who know about your health situation and help you out?”  “Have you ever noticed that how you feel emotionally affects your blood pressure or energy levels?” |
| Daily lifestyle | Explore readiness regarding smoking, alcohol, sleep, and home safety | “Have you ever thought about making any changes to your smoking (or drinking) habits?”  “How would you describe your sleep lately? Is it something you have wanted to improve?”  “Have you made any changes at home to make it safer for you to move around?” |

**Table A2.6b Contemplation Stage**

Core goal: Use decisional balance to help participants weigh the pros and cons of change. Elicit and amplify change talk. Explore ambivalence with curiosity, not persuasion.

| Domain | Core MI Goal | Representative Questions |
| --- | --- | --- |
| Blood pressure control | Strengthen motivation for monitoring; link to valued outcomes | “What do you see as the main benefits of monitoring your blood pressure regularly?”  “What gets in the way of doing it consistently? What would make it easier?”  “On a scale of 0–10, how important is it to you to keep your BP under control? What would need to happen to make it a 9 or 10?” |
| Medication management | Elicit self-articulation of reasons to take medication consistently | “You mentioned you take your medication most of the time. What do you think would happen if you stopped?”  “In your own words, what is the main reason you take your blood pressure medication?”  “What makes it hard to take it every day? What would help with that?” |
| Diet and nutrition | Weigh short-term preferences vs. long-term functional gains | “If you made some changes to your diet say, a bit less salt , what do you think you would gain? What might you lose?”  “What would it mean for your independence if your health improved over the next year?”  “Are there any healthy food changes you feel like you could realistically make?” |
| Physical activity | Resolve ambivalence about exercise; explore perceived consequences | “You mentioned thinking about exercising but feeling unsure. What is the main thing holding you back?”  “What is the best thing that could happen if you started doing some regular activity?”  “What kind of physical activity, even small, do you think you could enjoy?” |
| Psychological and social support | Strengthen awareness of social connection as a health resource | “How do the people around you support or not support your health habits?”  “What would it mean to you to feel less isolated or more supported in managing your health?”  “Is there one person in your life you feel comfortable talking to about how you have been feeling?” |
| Daily lifestyle | Guide reflection on benefits of behavior change for independence | “What would be the biggest benefit for you personally if you reduced your smoking or alcohol?”  “How do you think your sleep quality affects your energy and mood during the day?”  “What would need to change for you to feel more confident about your home being safe?” |

**Table A2.6c Preparation Stage**

Core goal: Collaboratively develop specific, feasible action plans. Participant takes the lead in decision-making. Plans should be closely aligned with the participant’s daily life, preferences, and functional status.

| Domain | Core MI Goal | Representative Questions |
| --- | --- | --- |
| Blood pressure control | Collaboratively develop a specific, feasible monitoring plan | “When would be the best time of day for you to measure your blood pressure? Morning, evening, or both?”  “What kind of BP monitor do you feel most comfortable using?”  “How will you keep track of your readings? Would a logbook, phone app, or something else work best for you?” |
| Medication management | Develop practical systems to overcome implementation barriers | “What reminder system would work best for you to make sure you take your medication every day?”  “If you start to feel a side effect, for example, dizziness when you stand up, what would you do?”  “When do you plan to next check your medication supply at home?” |
| Diet and nutrition | Build a realistic dietary change plan; anticipate challenges | “Which specific food change do you want to start with, reducing salt, adding more vegetables, or something else?”  “Who in your household does the cooking? How can you involve them in this plan?”  “What is one situation where you might find it hard to stick to your plan, and how could you handle it?” |
| Physical activity | Establish a concrete, safe exercise plan with gradual progression | “What type of exercise do you want to start with? How many minutes per day feels manageable right now?”  “Where do you plan to exercise, at home, in a park, or somewhere else?”  “What will you do on the days when you really do not feel like exercising?” |
| Psychological and social support | Agree on specific social engagement activities | “Which family member or friend would you feel comfortable sharing your health plan with?”  “Is there a community group or activity you have been thinking about joining?”  “How often would you like to try to make social contact, daily, a few times a week?” |
| Daily lifestyle | Develop concrete plans for smoking, sleep, alcohol, and home safety | “What is one specific step you will take this week to improve your sleep routine?”  “Are there any trip hazards at home you could remove this week? Who could help you?”  “If you were going to cut down your alcohol intake, what would the first step look like?” |

**Table A2.6d Action Stage**

Core goal: Provide timely, specific positive feedback. Help participants develop problem-solving skills.

| Domain | Core MI Goal | Representative Questions |
| --- | --- | --- |
| Blood pressure control | Affirm monitoring efforts; link readings to lifestyle factors | “I can see you have been measuring your BP consistently. How has that been going? Have you noticed any patterns?”  “Looking at your records from this week, what do you think might have influenced your readings?”  “How does it feel to be taking this kind of control of your health?” |
| Medication management | Identify practical barriers to adherence; problem-solve in real time | “Have there been any days this week when you forgot or chose not to take your medication? What happened?”  “How is the pill organizer working out for you? Did you encounter any issues?”  “What would you do if you were traveling and realized you left your medication at home?” |
| Diet and nutrition | Acknowledge healthy choices; support stable dietary routines | “Tell me about a healthy eating choice you made this week that you are proud of.”  “Were there any situations where sticking to your diet plan was hard? What did you do?”  “How are you finding the taste of food with less salt? Has that changed over time?” |
| Physical activity | Support maintenance of regular exercise; address early barriers | “How many times did you manage to exercise this week? How did it feel?”  “Was there a day you wanted to exercise but something got in the way? What happened?”  “Who in your life could exercise with you or encourage you to keep going?” |
| Psychological and social support | Reinforce value of social connection; strengthen emotional regulation | “Did you manage to connect with family or friends this week? How did that feel?”  “Have you noticed any change in your mood or stress level since you started engaging more socially?”  “What activity this week helped you feel most at ease or most yourself?” |
| Daily lifestyle | Affirm lifestyle improvements; reinforce positive bodily experiences | “Have you noticed any changes in how you feel since reducing your smoking (or alcohol)?”  “How has your sleep been this week? Did the bedtime routine you planned help?”  “Have you made any home safety changes? How do you feel moving around your home now?” |

**Table A2.6e Maintenance Stage**

Core goal: Consolidate internalized behaviors, prevent regression, and strengthen relapse-prevention plans.

| Domain | Core MI Goal | Representative Questions |
| --- | --- | --- |
| Blood pressure control | Reinforce role identity as active health manager; sustain under adverse conditions | “Monitoring your blood pressure has become part of your daily life now. How does that feel?”  “Have there been any days when it was harder to keep up your monitoring routine, like when you were not feeling well or were away from home? How did you manage?”  “Have you shared any of your BP management tips with anyone in your family?” |
| Medication management | Prevent self-discontinuation; consolidate long-term treatment commitment | “When you are feeling well and your BP is stable, do you ever feel tempted to reduce or stop your medication?”  “What reminds you why it is important to keep taking it even when you feel fine?”  “What is your plan if you ever go through a period when staying on your medication feels really hard?” |
| Diet and nutrition | Sustain healthy eating as internalized lifestyle; reframe lapses | “Your diet has changed a lot since we started. What do you notice about how your body feels?”  “Has there been a recent occasion, like a family meal, where it was hard to stick to your diet plan? What happened?”  “If you had a day where you ate more salt than planned, what would you tell yourself?” |
| Physical activity | Prevent boredom-related relapse; integrate exercise as valued routine | “You have been exercising regularly for a while now. Is there any type of activity you have been curious to try?”  “What keeps you motivated to keep exercising, what do you enjoy or value about it?”  “How has regular exercise changed how you feel about your body and your independence?” |
| Psychological and social support | Shift from support recipient to peer supporter; expand social contribution | “Have you ever thought about sharing what you have learned with other older adults in your community?”  “What would it mean to you to play a more active role in your community group or neighborhood?”  “How has your relationship with the people around you changed since you started working on your health?” |
| Daily lifestyle | Consolidate healthy habits as personal assets; extend healthy influence | “Looking back over these months, which health habit change are you most proud of?”  “How have your family members responded to the changes you have made at home?”  “If a friend told you they were trying to make the same changes you have made, what advice would you give them?” |
